# Response to commentary on “the middle fossa approach with self-drilling screws: a novel technique for BONEBRIDGE implantation”

**DOI:** 10.1186/s40463-019-0375-2

**Published:** 2019-11-05

**Authors:** Peng You, Lauren H. Siegel, Zahra Kassam, Matthew Hebb, Lorne Parnes, Hanif Ladak, Sumit Kishore Agrawal

**Affiliations:** 1 Department of Otolaryngology-Head and Neck Surgery, London Health Science Centre, Schulich School of Medicine & Dentistry, Western University, Room B1-333, London Health Sciences Centre – University Hospital 339 Windermere Road, London, Ontario N6A 5A5 Canada; 2 Department of Medical Imaging, St. Joseph’s Health Care London, Schulich School of Medicine & Dentistry, Western University, London, Canada; 3 Department of Clinical Neurological Sciences, London Health Science Centre, Schulich School of Medicine & Dentistry, Western University, London, Canada; 4 Department of Medical Biophysics, Schulich School of Medicine & Dentistry, Western University, London, Canada

**Keywords:** Bone conduction implant, BONEBRIDGE, Middle fossa approach, Conductive hearing loss, Surgical technique, Implants

## Abstract

The aim of this letter is to respond to a commentary on a published article on the middle fossa approach to BONEBRIDGE implantation with self-drilling screws published by the senior authors.

Dear Dr. Carnevale,

We appreciate your support of the middle fossa approach and your feedback on this paper. The senior author (S.K.A.) developed the middle fossa technique with self-drilling screws in 2012 to treat patients with mastoid cavities, and the first patient was implanted following this approach in April, 2013 at the London Health Sciences Centre [1, 2]. This particular technique was previously published [3, 4], and the current series examines the long-term results (40 patients, up to 71 months follow-up).

It is exciting that your group subsequently adopted a similar surgical technique (14 patients, up to 45 months follow-up). Furthermore, it is reassuring that surgical and audiologic outcomes were similar between centres, regardless of whether self-drilling or self-tapping screws were used. We apologize that we were unable to cite your results within the current paper. Unfortunately, our manuscript was completed for submission prior to the publication of your paper in April, 2019.

To the best of our knowledge, the use of a neurosurgical perforator or trocar for the BONEBRIDGE was first published by Barbara et al. in 2013 [5], albeit in the retrosigmoid approach. As mentioned in our paper, we also used the neurosurgical perforator in our first few middle fossa patients. Although use of the perforator was quick to create the initial craniotomy (14 mm outer drill and 11 mm inner drill), expansion of the craniotomy to 16 mm to fit the BONEBRIDGE took additional surgical time. In your paper (and supplementary video), initial craniotomoy using the perforator took 14 s, however the total craniotomy time including enlargement with the Kerrison rongeur was not described.

Neurosurgical perforators are associated with complications [6, 7], and have significantly higher costs than regular otologic drills [8]. Use of otologic drills to create the craniotomy is a safe and efficient alternative, and neurotologists comfortable with the middle fossa approach for acoustic neuromas should be familiar with this method. Therefore, the senior authors (L.S.P. and S.K.A.) opted to exclusively switch to otologic drills, and no adverse events or significant increases in operative time were noted.

## Data Availability

Not applicable
